# Medical News and Miscellany

**Published:** 1895-05

**Authors:** 


### Medical News and Miscellany.

---

**Dr. Sam W. Hart**, of Mineola, charged with killing Charles Apel, was acquitted May 8th.

---

Our friend Dr. H. H. Darr, of Caldwell, lost his office by fire, on the night of April 30th, ult.; loss, $500.

**At a late** meeting of a certain medical society in Texas, a certain measure was proposed, and it is said the man who "kicked" most was a *one legged doctor.*

---

**The** Commencement Exercises of the fourth session of the Medical Department of the University of Texas will take place Wednesday evening, May 15th, 1895, at 8 o'clock, at the Opera House, Galveston.

---

**A Large Enterolite.**—Dr. Wylie, of New York, recently removed a stone two inches long by an inch wide, from the intestinal tract, at the ileo-cecal valve. The stone had produced obstruction of the bowels.

---

**The Austin District Medical Society** will hold its next quarterly meeting on the 20th of June. A full and interesting programme will be mailed to members in due time. All regular physicians accessible to Austin are cordially invited.

---

**The** third International Congress of Dermatology will be held in London, August 4th to 8th, inclusive, 1896. Mr. Jonathan Hutchinson is President. The Vice-Presidents for the United States are Duhring, White (of Boston), Nevins-Hyde, Bulkley, Keys, and Fox.

---

**A Rival.**—Bill Nye says he is very apprehensive that the pleasant relations heretofore existing between him and his brother millionaire Vanderbilt, whose "ranch" in North Carolina adjoins his, are to be interrupted, and the beginning of a feud, which will become famous he says;—will even rival the celebrated Mellin's "feud."

---

**We** have received from the Rio Chemical Co., of St. Louis, a complete map of the world, corrected up to date. Size of map, 27x27 inches, mounted, ready for hanging. This is really a very accurate and convenient map, and will be useful to any physician. If you have not already received one, write to the Rio Chemical Company, St. Louis, Mo. They will be pleased to send it to you, prepaid.

---

**The Young Governor** says he is a friend to education, but he has, nevertheless, vetoed every appropriation for its advancement that could be reached, to-wit: $6000 for electrical plant for University, and $5000 to keep up the library, and some other items; and with regard to the Medical Department, he vetoed the allowance of $2000 for each year, 1895 and 1896, for salary of instructor and assistant for the training school for nurses. This latter the JOURNAL regrets very much, as it cripples,—forestalls in its infancy—a movement which, if fostered, would be productive of much good,

---

**Fort Worth Medical School,** or more properly the Medical Department of the University of Fort Worth, held its first annual commencement on April 25th, ult. There was an interesting program for the occasion, and the JOURNAL regrets its inability to be represented on the occasion. A write up of the event was promised us, but to date it has not been received. The following young men received the degree "M. D.": Thomas Jones, Tip M. Collins, Nicholas L. Dudley, Alvin B. Minton, John W. James, Benjamin F. Loring, William N. Wardlow.

---

**Excursion to Monterey and Mexico, via International Route.**—A special excursion train will leave San Antonio on the morning of June 6, 1895, for Monterey and Mexico City. Rates, San Antonio to Monterey and return, $5.00, and to Mexico City and return, $20.00, tickets limited to fifteen days for return. Stop-over will be allowed at all points in Mexico. Call on nearest agent I. & G. N. R. R. for full information, or address

D. J. PRICE, A. G. P. A., I. & G. N. R. R.,
Palestine, Texas.

---

**We** have recieved from Dr. James E. Reeves, of Chattanooga, Tennessee, author of "Medical Microscopy," two valuable mounted microscopic slides, one of them being a section of inguinal gland from a Hong Kong Bubonic Plague patient, and the other a section of an anthrax lung from a guinea pig. The section of inguinal gland shows up beautifully the little bacillus with rounded ends described by Kitasato and Yeisen. Both of the sections are beauties, and should any of our readers desire to possess duplicates of either or both slides, they can be obtained from Dr. Reeves at $1 each.

---

**A. M. A., Baltimore.**—Telegram to TEXAS MEDICAL JOURNAL, Austin: Officers for ensuing year—Dr. R. Beverly Cole,

California, President; Dr. J. J. Chisholm, Maryland, First Vice-President; Dr. J. E. LeGrande, Alabama, Second Vice-President; Dr. A. B. Clark, Massachusetts, Third Vice-President; Dr. T. Sutler White, Kansas, Fourth Vice-President; Dr. Frank Woodhite, Pennsylvania, Secretary. Association will meet next year at Atlanta, Georgia. H.

The JOURNAL congratulates Brother LeGrand, of the *Alabama Age*. But what's the matter? they swapped off, or let go, the invincible Atkinson, so long time Secretary.

---

**A Substitute for Laceration of the Perineum.**—The venerable Dr. W. A. Morris, of Austin, some years ago, wrote an article in which he advocated the making of a bilateral incision in the vaginal rim in cases where a posterior laceration was considered inevitable. The paper was based upon an experience in several cases treated in this manner, and claimed the following points of advantage:

1. Only a nick in the rim on both sides, a quarter of an inch deep, made when the tissues are tense, is all that is required.

2. The incisions, when made, are easily repaired by a few stitches.

3. The danger of sepsis is much lessened by the track being sound over which the discharge passes, the wounds being above, out of the way.

The above suggestion has never received the attention which it deserves.

---

**The Chair of Pharmacy** in the Medical Department, University of Texas, made vacant by the resignation of Prof. Jas. Kennedy, since deceased, will have to be filled by the Regents next month. The selection of a successor is very important, and the claim of every candidate should be and will be closely investigated. There are several aspirants, amongst them the JOURNAL learns is Dr. L. Myers Connor, of Dallas. Dr. Connor is very strongly endorsed. He is the originator of the Texas State Pharmaceutical Association, and is its President. Not only is he endorsed by his Texas constituents, but will present, we are informed, a very strong testimonial from Dr. Wm. Simpson, President of the American Pharmaceutical Association, Raleigh, N. C.; one from Prof. Robt. G. Eccles, the famous chemist of Brooklyn, and one also from Prof. C. S. Halleberg, Director of the National Institute of Pharmacy, Chicago. *Cæteris paribus*, we would prefer a

"home" man for the place, and nominate Dr. Connor. Dr. Eagon vouches for him also, and that is sufficient guarantee.

---

**Governor Culberson** vetoed the bill which took the appointing of asylum superintendents out of the hands of the board of managers and placed it directly in the hands of the governor, but restricting the appointment to physicians who have had not less than two years' experience in asylum management. He said:

"The bill too narrowly limits the number of physicians from whom selections may be made. * * * * Nor is it deemed wise to limit the appointment of superintendents to physicians who have specially treated the insane. These superintendents perform other and equally responsible duties as those of mere attending physicians. They care for great properties, direct the economical expenditure of more than $100,000 annually, control and manage a large retinue of employes and servants, and should be selected as well for their executive and administrative capacity, as for their professional skill and learning."

What's the matter with making the selection from the trades and arts? say a foreman of a big factory, or something of the kind; why a doctor?

---

### Death of Dr. Cupples.

[From the San Antonio Express.]

After an illness of twenty-one days Dr. George Cupples, at 4 o'clock, Friday morning, April 19th, succumbed to the ravages of time, and passed away at the ripe old age of seventy-nine years.

Up to the very day when his last illness seized him, Dr. Cupples attended to his large professional practice, and the news of his death was a great shock to his host of friends, who did not even know of his illness. He met the angel of death as he met every trial, danger, duty and responsibility in life, calmly, resolutely, fearlessly and philosophically, a knightly Christian gentleman.

In the death of Dr. Cupples San Antonio loses one of its most distinguished and honored citizens, one who has been closely identified with every interest of the city for more than half a century. He was among the first physisicians to locate in San Antonio, and for many years he and Dr. Ferdinand Herff were about the only physicians of note in this section. Everybody who knew Dr. Cupples was his friend, and his death has cast a shadow of gloom over the entire city.

The professional attainments of Dr. Cupples were of the highest order. His record and early training were such as few men enjoy, and the many important official positions he has been honored with in the line of his profession attest a recognition of these facts. Dr. Cupples was a remarkable man in many respects, a man of strongly marked individuality of character, and yet not possessed of those eccentricities that are often found in men of genius or exceptional abilities. Modest and gentle as a woman, thoughtful to the verge of gravity, dignified almost to austerity, a refined nicety of manner and expression upon all occasions, gave such a caste and tone to the entire man that to those who possessed neither the time, patience, ability or opportunity to penetrate beneath that urbane and placid exterior he seemed too scrupulously particular to meet and deal with the practical professional problems requiring the exercise of energy, promptness, nerve, skill and strong common sense. But no man who knew him well enough to form an intelligent opinion ever fell into this erroneous estimate of his character. To his eminent skill and learning as a practitioner, acquired in the Universities of Edinburgh, London and Paris, he added the vast knowledge acquired in an active practice and in a field unusually rich with opportunities.

At the grave Dr. Frank Paschal, a lifetime friend and pupil of the deceased, delivered a short oration. He said:

"SORROWING FRIENDS AND COLLEAGUES:—Your sad faces and bowed heads speak the feeling you have for Dr. George Cupples, and there is little need for words to express the reasons why you hold his memory dear; his deeds live with you and are far greater to tell than are words.

"The life of Dr. Cupples makes one of the most beautiful pictures that the mind can conceive. A life full of honor, humanity, love and self-sacrifices; a life given to mankind for more than fifty years, and all because he loved his fellow-men. And he loved his work with all his heart, his life, his soul. With him, to do good, was to do his duty, and it came spontaneously from his heart.

"His brain was a storehouse of information, not alone in medicine, but in everything that goes to make man great in the possession of knowledge, and it was his ability to apply this knowledge that made him a giant among men. No one could be long in company with him without feeling that they had learned a great deal, and each hour spent with Dr. Cupples taught those whose privilege it was to associate with him, new lessons, and none better than the members of our profession knew how to appreciate the wisdom of this grand old man, and rely upon his advice and judgment.

"It was given to Dr. Cupples, that which is allotted to but few, that he should retain his mental faculties in their full strength in his declining years, and thus enable him to do the grand work for which he had so ably fitted himself until his last brief illness called him to his eternal rest.

"It was not for sordid gain that Dr. Cupples practiced his profession, for had it been, affluence and wealth would have surrounded him long before his summons came; it was for the love of his profession, and the good that he could do, that made this great philanthropist battle with disease. And the call for mercy, whether from the most humble or the greatest, met with the same willing and prompt response.

"The dangers that beset him from an unsettled condition of this country in his first years of practice, and the dangers from disease weighed as naught; but strong in the feeling that he was doing his duty, he carved his name among those of the great heroes.

"He was always abreast with the advances of his profession; was the first physician in this State to use an anæsthetic, and was always foremost to satisfy himself of the value or worthlessness of new remedies or discoveries. He was a brilliant surgeon, possessing every attribute that makes the surgeon, and when his mind was assured of the necessity of the knife, his fertile brain guided his skillful hand with unerring success. As a sanitarian he was ever zealous to advise and counsel for the welfare of the State and community, and no one worked harder, or was more interested in providing for the proper sanitation of this city.

"He was relentless in his efforts to secure medical legislation that would protect our people against frauds and impostors, and his last work was a bill that he drafted about a month before his death, but which he failed to see become a law. Could he speak, he would tell his colleagues that they could pay him no greater tribute than to take up the work that he laid down, and carry it on until the State, that he loved so dearly, is placed in the foremost rank in medical advancement and medical civilization.

"As a citizen he loved law and order, and respected the right of others, and gloried in the prosperity of his adopted country. As a friend he was kind, generous and true, and there are many who remain to tell of his acts of friendship. His life work attested to his Christianity, and drew him in sweet communion with his Maker.

"Those who knew the worth of this noble man will long mourn him. His ministrations and assuring words of hope and encouragement to the multitude of sufferers will be sadly missed, but his acts that made him great will always remain in the mems ory of a grateful people, and their tears will water his grave."

The active pall bearers were Dr. Frank Paschal, A. W. Houston, Gen. W. H. Young, Dr. J. V. Spring, Dr. E. R. King and L. Orynski. The honorary pall bearers were Mayor Henry Elmendorf, Dr. G. G. Watts, Dr. D. Berry, Col. Elias Edmonds, Dr. A. S. McDaniel, Dr. F. M. Hicks, Leonard Garza, Dr. B. F. Kingsley, Dr. L. L. Lacey.

The Southwest Texas Medical Association and the San Antonio Pharmaceutical Association attended in a body.

The remains were interred in the San Fernando cemetery besides those of his first wife.

### RESOLUTIONS OF RESPECT.

At a called meeting of the West Texas Medical Association, held last night, the following resolutions were unanimously adopted:

WHEREAS, The Divine Ruler of the Universe has seen fit to remove from among us Dr. George Cupples, our friend and the founder of the West Texas Medical Association, as well as its first president, who was the pioneer of organized medicine in the State of Texas, a leader and adviser, who seldom if ever erred; therefore be it

*Resolved*, That in the death of Dr. Cupples, not only we, but the whole profession have lost an honored and faithful co-laborer, and the community has cause to mourn the loss of a skillful and learned physician, an honest and sympathizing friend.

*Resolved*, That by his kindness of manner, by the thoughtful interest he always manifested in the younger members of the profession, by his encouragement, his earnestness and his example he had endeared himself to all, and that if we would fitly honor and cherish his memory we must emulate his zeal and vie with each in carrying forward the great work he loved so much, and in which he was ardently engaged.

*Resolved*, That we tender to his family in this sad hour of affliction our heartfelt sympathy.

*Resolved*, That these resolutions be handed the family of our deceased brother, and to the daily papers and to the TEXAS MEDICAL JOURNAL, and be spread upon the minutes of this Association.

JOHN V. SPRING,
FRANK PASCHAL,
C. E. R. KING,
G. G. WATTS,
D. BERRY,
Committee.

At a called meeting of the San Antonio Druggists' Association, held last night, the following resolutions were adopted:

WHEREAS, It has pleased an all-wise Providence to remove from our midst by death Dr. George Cupples; therefore be it

*Resolved*, That the San Antonio Druggists' Association, while bowing with resignation to the will of the Almighty, do learn with sorrow of the demise of that distinguished citizen and illustrious member of the medical profession.

*Resolved*, That the City of San Antonio in the death of Dr Cupples has lost one of its most respected, venerable and upright citizens, and one who stood by the city in time of need, and whose many good deeds will perpetuate his memory and be more lasting than monuments of marble.

*Resolved*, That the medical profession has lost one of its brightest ornaments, but to his record it can always point with pride, which stands out in bold relief and challenges the admiration of mankind, and will be a shining model and beacon light for the present and future generations yet unborn.

*Resolved*, That the San Antonio Druggists' Association have lost a loyal friend, patron and supporter, one who by his wise counsel and example gave spirit and emulation to our Association.

*Resolved*, That the public at large have lost a benefactor, both as a citizen and physician, whose genial and helping presence and skill will ever be missed.

*Resolved*, That we extend our heartfelt sympathies to his bereaved family in this the sad hour of their affliction.

*Resolved*, That a copy of these resolutions be furnished his family and printed in the city papers.

GEORGE J. F. SCHMITT,
GEORGE H. KALTEYER,
C. SCHASSE,
A. DREISS,
E. REUSS,
W. R. CLAVIN,
Committee.

### Medical Advertising and the Mails.

NEW YORK, March 28, 1895.

*To the Editor of the New York Medical Journal:*

SIR:—I received to-day (in common with many other victims, I suppose) a pamphlet containing some twenty reproductions of photographs of syphilitic skin lesions. Many of the pictures were of men stark naked, and showing uncommonly well-developed genitalia. One picture showed the genitalia, and but little of the man.

This pamphlet, issued to advertise some proprietary nostrum, was sent by mail to my house, and lay for a while on the table of my waiting room in an *unsealed* envelope, so that any woman or child prompted by curiosity might easily have inspected it, which they would have been all the more prone to do since the wrapper was marked in large letters, "for medical men only."

While photographs of this kind have a legitimate place in systematic works on syphilis, to use them for purposes of commercial exploitation is disgusting, and to thrust them into a man's house in such a way that any member of his family—the women, the children, and the female servants—may easily get hold of them, is a signal invasion of his rights.

I have sent the pamphlet, with the wrapper as I received it, to the Postmaster-General at Washington, with the request that the postoffice take means for the abatement of this nuisance. For scarcely a week goes by without there arriving by mail, in open envelopes, pamphlets marked in large letters on the covers, "Painful Menstruation," "Sterility," "Impotence," or some such title calculated to arouse curiosity, and it is some trouble to be obliged to tear these to pieces small enough to be useless before throwing them into the waste basket. But pamphlets of this sort are petty annoyances compared with the ostentatious indecency of the publication received to-day.

While we may call on the postal authorities to protect us from having these things thrust into our houses in unsealed envelopes, yet the ultimate cure for this evil (steadily on the increase) must be sought in the attitude of the medical profession itself, which should mete out a stern disapproval to all who, for the sake of selling their wares, offend against decency and good sense.

WALTER MENDELSON, M. D.

NEW YORK, March 29, 1895.

*To the Editor of the New York Medical Journal:*

SIR:—A letter has reached me from a medical practitioner of this city in which he complains regarding a pamphlet recently sent out by our company. This pamphlet is intended to show the therapeutic value of arsenauro and mercauro. It is entitled "Twenty Photographs for the Medical Profession Only," and it means exactly what it says. My response to the complainant will be found herein. May I ask that you publish it?

CHARLES ROOME PARMELE.

[Mr. Parmele's inclosure.]

"NEW YORK, March 28, 1895.

"—— ——, M. D.,

"—— *Street, New York:*

"DEAR SIR:—Your favor of even date just received. If our pamphlet has reached other eyes than your own in your house, the writer exceedingly regrets the fact. Upon both the envelope and the pamphlet are the plain Anglo-Saxon words, 'For the Medical Profession Only.' Far worse illustrations appear in the medical journals and in medical text-books, and if you subscribe for any you must know that neither reach you in sealed envelopes. It occurs to us that every physician who keeps pace with medical progress must constantly receive in his office many things which are profitable to him, yet not intended as an amusement to the household. The writer claims to possess proper instincts as to morality, propriety, and refinement; hence the wording on the envelope and on the pamphlet.

"You speak of our goods as nostrums. Permit us to say that they are not such; they are very remarkable chemical products and indorsed by professional colleagues of yours whose utterances we do not believe you will have the temerity to question. We do not permit the laity to have any of our literature, our work being done in strictly ethical channels.

"Pardon us if we offer the suggestion that, inasmuch as your office and residence are in the same building, you do as other medical practitioners do—namely, insist that communications addressed *to you* be opened *by you* and *not* inspected by those to whom the communications are *not* addressed. If the members of a household should inspect every medical journal which comes to a physician, your line of argument would exclude the very data which make medical journals of value. How about the *New York Medical Journal*, the *New York Journal of Gynæcology and Obstetrics*, the *American Journal of Obstetrics*, the *Journal of Cutaneous and Genito-urinary Diseases?* All of them contain illustrations of interest to a physician, but *not* of profit to his family. Very truly yours,

"CHARLES ROOME PARMELE."

[The above is reproduced from the *New York Medical Journal* as being of interest to all physicians, and especially as there are differences of opinion among physicians on the subject. There are two sides to this question, as there are said to be to all questions; and undoubtedly, in our opinion, Mr. Parmele is on the correct side. He might have asked his doctor-correspondent if his family do not have access to his medical library? Many physicians keep most of their medical books at home, for night reading, and if he were not afraid Susan or Jane, or some member of his family, might get hold of *Morrow's Atlas*, for instance. Physicians who will allow the average daily paper, and we blush to say it, especially the religious papers, to come into their families, with all the vile stuff they publish—pictures and all—ought not to complain of strictly medical works; for they—the papers—are infinitely worse; they put evil thoughts in the minds of young persons which would never be suggested by any such pictures as are described; they incite to immorality. For instance; a lady is *assured* of immediate relief—for suppression, but is cautioned not to take the medicine if pregnant! *An abortifacient* recommended as *sure* to produce the desired effect.

Why not ask Mr. Postmaster Wilson to taboo Gray's Anatomy? Out on such prudery.—ED.]

### A Texas Doctor in Luck.

Friends of Dr. L. W. Cock, late of San Marcos, Texas, will be pleased to learn that he has perfected a device for disinfecting, and for preserving meats, etc. It is an ingeniously constructed apparatus which burns sulphur, boracic acid, and some other ingredients, liberating a gas which has been said to be a pure bisulphide of carbon. This gas, while deadly to all germ, insect and parasite life,—and even to higher organizations, rats, mice, roaches, ants, etc., is said to be free from danger to man. The doctor is in Chicago; has organized a stock company, and has gotten all the pecuniary assistance necessary to introduce his invention. He writes the JOURNAL that experimental tests have been made which show it is especially well adapted to disinfecting hotels and sleeping cars. In his pamphlet the doctor very pertinently says:

"Why should you be required to occupy a room at a hotel, a berth in a sleeping car, or a state-room in a steamer, unless the same has been disinfected, when it may possibly have been occupied the night before by a consumptive or a person suffering from small-pox, diphtheria, yellow fever, typhoid or scarlet fever, or a score of other diseases? Why, we ask, should this be required of you, any more than that you should be required to sleep between the same sheets his body has polluted—when for a few cents the apartment can be fumigated with the Acme, which will insure safety and cleanliness.

"Surely, the prices charged and paid for those accommodations are large enough to warrant the expenditure of five or ten cents to rid the room of all disease germs, and guarantee the guest or occupant an atmosphere to stay in, as pure and sweet as that in the mountains."

The doctor is right

